# Does Preventive Care Reduce Severe Pediatric Dental Caries?

**DOI:** 10.5888/pcd17.200003

**Published:** 2020-10-29

**Authors:** Helen H. Lee, Luis Faundez, Kamyar Nasseh, Anthony T. LoSasso

**Affiliations:** 1 Department of Anesthesiology, College of Medicine, University of Illinois at Chicago, Chicago, Illinois; 2 Department of Economics, University of Illinois at Chicago, Chicago, Illinois; 3 Health Policy Institute, American Dental Association, Chicago, Illinois; 4 Department of Economics, DePaul University, Chicago, Illinois

## Abstract

**Introduction:**

Tertiary oral health services (caries-related surgery, sedation, and emergency department visits) represent high-cost and ineffective ways to improve a child’s oral health. We measured the impact of increased Texas Medicaid reimbursements for preventive dental care on use of tertiary oral health services.

**Methods:**

We used difference-in-differences models to compare the effect of a policy change among children (≤9 y) enrolled in Medicaid in Texas and Florida. Linear regression models estimated 4 outcomes: preventive care dental visit, dental sedation, emergency department use, and surgical event.

**Results:**

Increased preventive care visits led to increased sedation visits (1.7 percentage points, *P* < .001) and decreased emergency department visits (0.3 percentage points, *P* < .001) for children aged 9 years or younger. We saw no significant change in dental surgical rates associated with increased preventive dental care reimbursements.

**Conclusion:**

Increased access to preventive dentistry was not associated with improved long-term oral health of Medicaid-enrolled children. Policies that aim to improve the oral health of children may increase the effectiveness of preventive dentistry by also targeting other social determinants of oral health.

**Summary**

**What is already known on this topic?**

Increasing dental provider reimbursements for preventive care visits is an effective policy intervention to increase preventive dental visit use among Medicaid-enrolled children.

**What is added by this report?**

Increased preventive care dental visits did not translate into significant changes in pediatric dental surgeries after a reimbursement-focused policy intervention.

**What are the implications for public health practice?**

Interventions to reduce pediatric dental surgery should reflect the social determinants of oral health, including access to regular dental care and household oral health behaviors.

## Introduction

Dental caries is the most common childhood chronic disease in the United States and worldwide. It disproportionately affects vulnerable children such as those who are poor, receiving Medicaid, of a racial/ethnic minority group, or residents of an underserved area (1,2). The imbalance in incidence of severe caries reflects disparities in oral health care access and use and other social determinants of oral health. In this context, some children among those at high risk for caries develop such severe disease that they seek care in the emergency department (ED) or require dental surgery under general anesthesia (DGA), both of which are costly and often ineffective interventions (3,4).

Clinical care alone does not address the multifactorial causes of caries. Oral health behaviors, caregiver psychosocial state and parenting style, dietary choices, health literacy, and fluoride exposure are only a few factors that influence a child’s oral health (5–10). However, policy interventions to improve access to preventive care have focused on provider reimbursement (11), which may be of limited effectiveness in improving access to preventive care, particularly in states with relatively high reimbursement levels or high numbers of dentists participating in Medicaid programs (12). Additionally, although reimbursement may affect getting children to the dental office, it may not influence services received there and may differentially benefit the oldest children rather than the youngest (13). In response to a 2004 US Supreme Court decision that required Texas’s Medicaid program to comply with guidelines on increasing access to dental care providers (14), Texas increased the amount of fees reimbursed for dental preventive care by 52.5% on September 1, 2007.

Although increasing access to preventive dental care is important, preventive care alone does not reduce the likelihood of needing tertiary oral health services (caries-related surgery, sedation, and ED visits) (15). Previous research established that Medicaid reimbursements for preventive dental care substantially increased self-reported preventive dental visits in several states (11). However, increasing Medicaid reimbursements to dental care providers for preventive services has not substantially decreased disparities in pediatric oral health related to age, race/ethnicity, and income (16). Medicaid reimbursements can improve access to and use of preventive dental services for some Medicaid beneficiaries, but how this translates into improved oral health outcomes for the population with the highest disease burden — children who require dental procedures with anesthesia — is unknown. Additionally, the clinical efficacy of increased preventive care dental visits to reduce severe caries in early childhood is questionable, because risk factors (oral health behaviors, cultural oral health beliefs) encompass social determinants of health that operate on individual, community, and environmental levels (17). Our study extends knowledge of the effectiveness of a prevention-aimed policy to improve long-term oral health outcomes. Furthermore, ours is the first study to suggest causality between increased preventive dental care and changes in use of tertiary oral health services.

## Methods

We studied children aged 9 years or younger who were enrolled in the Texas and Florida Medicaid programs. Texas children were the treatment group, and Florida children were the control. Florida was selected as the control because its trends in reimbursement for preventive dental care were stable during the study period as a ratio of private insurance to Medicaid rates (11).

The estimated prevalence of DGA in the Medicaid population during our study period was ~0.5% to 1% (18,19). A dental surgical event was the primary outcome of interest. In a sample size calculation, assuming α = 0.05, β = 0.05, and power = 0.95, we estimated the ability to detect a difference of 0.1 between treatment and control states in a sample size of 489,102 (20).

Patient demographics and outcomes were derived from Medicaid enrollment and claims files, which we obtained from the Centers for Medicare and Medicaid Services Research Data Assistance Center. Because of budget constraints, we limited our requests for data files for treatment and control states to the pre-reform (2007) and post-reform years (2011 and 2012). This period was selected to reflect prior work that evaluated the impact of these natural experiments on use of preventive dental care (11) and thus allow for the ability to compare changes in outcomes related to tertiary oral health services. This study was granted approval under expedited review by the University of Illinois at Chicago’s institutional review board (#2016–0573).

The validity of our findings was threatened by omitted variable bias. If changes in dental services reflect general trends in use of health care services, omission of these unknown variables would result in incorrectly attributing change to policy interventions (ie, increased reimbursements for preventive dentistry). To address this, we measured the effect of increased reimbursements for preventive dental care on appendectomies as a falsification test. If dental surgeries and appendectomies were associated with increased reimbursement for dental care in similar fashion, we would interpret this to signify confounding factors that influence both dental surgeries and preventive dental care reimbursement levels.

**Data management**. Outcomes were identified on the basis of American Dental Association Code on Dental Procedures and Nomenclature (CDT) (21) or the American Medical Association Current Procedural Terminology (CPT) codes (22) and *International Classification of Diseases, Ninth Revision, Clinical Modification (ICD-9-CM)* (23)*.* Surgical cases were identified by a combination of a general anesthesia claim with either the CDT or CPT coding system and a caries-related procedure claim (24), or a combination of a diagnosis of appendicitis and a CPT for an appendectomy. Procedures under sedation were similarly identified. Caries-related emergency department visits were identified by combining ICD-9 codes for nontraumatic dental conditions.

This observational study used difference-in-differences models to establish causal inference between the intervention (policy change) and outcomes. We assumed that any changes in use of dental general anesthesia would not be immediate and theorized that the largest changes in surgery would overlap with children at risk for early childhood caries, because most dental surgeries among Medicaid enrollees occur in children aged 1 to 5 years (19).

We used a linear regression model to estimate 4 outcomes: receipt of a preventive care dental visit, caries-related dental sedation visits, DGA, and an emergency department dental visit. State fixed effects accounted for time-invariant aspects of each state’s environment related to outcomes. To control for the possibility that other changes between 2007 and 2012 in the treatment state (Texas) could confound the effect of the Medicaid reforms on use, we used Medicaid-enrolled children from Florida, where no known policy changes occurred during that same period, as a comparison group. The econometric model used for each outcome was as follows:

Y = β_0_ + β_S_+β_1_Year_2011/12_ + β_2_TX *Year_2011/12_ + X + error

The vector X includes control variables (age, sex, race/ethnicity). Although the control variables are known to be important correlates to receipt of DGA and emergency department visits, the main variables of interest are the year by state interaction effects. For example, β_2_ represents the change in DGA in Texas after the Medicaid policy change took effect. The coefficient vector (β_S_) represents state fixed effects, which adjusts for varying outcome rates across states.

## Results 

A total of 7,748,850 children met study inclusion criteria. Demographic differences between Texas and Florida were primarily based on race/ethnicity, because approximately 60% of Medicaid enrollees in Texas were Latino, compared with approximately 30% Latino in Florida (Table 1). Use of all types of visits, unadjusted, increased in Texas between pre- and postpolicy periods: preventive care visit rates increased about 12 percentage points (24% from baseline); dental surgery rates, 0.2 percentage points (14% from baseline); sedation visits, 1.3 percentage points (40% from baseline); and emergency department visits, 0.09 percentage points (22% from baseline).

**Table 1:** Characteristics of Children Enrolled in Medicaid in Prepolicy (2007) and Postpolicy Periods (2011-2012), Florida and Texas

Characteristic^a^	Control: Florida		Intervention: Texas	
	Prepolicy	Postpolicy	Prepolicy	Postpolicy
**Total, no.**	591,584	2,146,677	1,032,194	3,978,395
**Age, mean, y**	3.5	4.0	4.1	4.0
**Race/ethnicity^b^**				
White	29.8	27.9	18.0	15.7
Black	27.2	28.4	14.9	12.9
Latino	32.8	31.9	64.3	58.6
Missing	9.2	10.5	1.3	11.4
**Female**	48.1	48.6	48.7	48.8
**Preventive care visits**	22.5	27.1	50.5	62.8
**Dental surgery**	0.3	0.5	1.4	1.6
**Dental sedation events**	1.0	0.9	3.2	4.5
**Emergency Department visits**	0.4	0.7	0.4	0.5

a Values are percentages unless otherwise indicated.

b We did not include the following race categories: American Indian or Alaska Native, Asian, and Native Hawaiian or Other Pacific Islander; therefore, race percentages will not total 100%.

Reimbursements for preventive care, sedation, general anesthesia, and other caries-related treatment services increased over the study period in Texas (Table 2). Reimbursements for preventive care and general anesthesia provided by medical anesthesiologists (CPT = 00170) did not increase in Florida over the study period.

**Table 2:** Reimbursement Rates for Preventive Care, Surgery, Sedation, and Emergency Department Visits Related to Dental Caries, Florida and Texas, 2007, 2011, 2012

Reimbursement Codes	Control State				Policy Intervention State			
	Florida, $			Change Post/Pre, %	Texas, $			Change Post/Pre, %
	2007	2011	2012		2007	2011	2012	
**Preventive care** a								
D0120	114.5	107.1	108.0	−6.1	22.8	31.4	31.2	37.3
D0150	89.8	88.0	80.5	−6.2	27.4	37.1	36.7	34.7
**General anesthesia**								
00170^b^	137.0	125.0	132.0	−6.2	154.0	253.0	254.0	64.6
D9220^a^	56.0	72.0	83.0	38.4	87.0	185.0	186.0	113.2
**Sedation** a								
D9241	50.0	62.9	73.5	36.4	101.9	118.8	120.7	17.5
D99143	61.6	100.1	102.8	64.7	59.5	82.1	148.7	93.9
D9248	40.0	50.0	58.9	36.1	144.6	182.8	182.9	26.5
**Dental procedures** a								
D2140	31.1	38.4	46.0	35.7	42.9	63.1	62.8	46.7
D2930	68.0	84.6	100.7	36.3	105.0	153.1	152.7	45.6
D7140	27.0	33.5	39.9	35.9	46.1	66.3	66.1	43.6

a American Dental Association Code on Dental Procedures and Nomenclature (CDT) (2011–2012) codes were used to identify reimbursement rates for services (21).

b American Medical Association Current Procedural Terminology (CPT) (2011) (22) codes were used to identify reimbursement rates for services.

To isolate changes in use in the policy intervention, we employed a difference-in-differences study design. We found that use changed significantly for all difference-in-differences outcomes (Table 3) when we controlled for time-invariant aspects of each state’s environment that related to outcomes and other possible changes between 2007 and 2012. First, we estimated the effect of the policy on all children aged 0 to 9 years. In the postpolicy period, preventive dental care visits increased 11.4 percentage points (*P* < .001, standard error [SE], 0.0008) from a baseline of 50.5%. The policy-associated increase in preventive care dental visits accounted for 93% of the overall increase in such visits. Dental surgery use increased by 0.01 percentage points (SE, 0.00016) in association with the policy intervention (baseline prevalence of 1.43%), without significance. However, surgery for a medical indication (appendicitis) increased significantly by 0.01 percentage points (*P* < .01; SE, 0.00004). Sedation visits increased by 1.7 percentage points (*P* < .001, SE, 0.0003) from a baseline of 3.2%. Emergency department visits for caries decreased in the postpolicy period by 0.3 percentage points (*P* < .001; SE, 0.0001) from a baseline of 0.41%.

**Table 3:** Effect of Policy on of Outcomes of Dental Care and Nondental Care Among Study Group (N = 7,748,850), Results for Difference-in-Differences Models^a^

Linear Regression Model	Preventive Visits	Dental Surgery	Sedation	Emergency Department	Appendectomy
Prepolicy, Texas	0.22^b ^(0.000684)	0.00984^b ^(0.000139)	0.0154^b^ (0.000217)	0.00109^b ^(0.000104)	−0.00001 (0.000039)
Postpolicy, Texas	0.021^b^ (0.000569)	0.00201^b ^(0.0000870)	−0.00345^b ^(0.000145)	0.00352^ b^ (0.0000978)	0.0000283 (0.0000316)
DiD	0.114^b^ (0.000764)	0.000104 (0.000160)	0.0172^b^ (0.000250)	−0.00273^b ^(0.000122)	0.000108^c ^(0.0000439)
Controls^d^	Yes	Yes	Yes	Yes	Yes
Observations, no.	7,748,850	7,748,850	7,748,850	7,748,850	7,748,850

Abbreviation: DiD, difference-in-differences model.

a Values are percentage (robust standard error) unless otherwise indicated. Policy impact estimated by looking at the difference-in-differences in outcomes using adjusted linear regression models. Utilization outcomes in Texas (intervention state) and Florida (control state) were estimated for the prepolicy period (2007). Prepolicy outcomes estimates are displayed only for the intervention state (Prepolicy, Texas). Outcomes were estimated in the postpolicy period, 2011-2012 and are displayed only for Texas (Postpolicy, Texas). The difference between pre-and postpolicy estimates between the intervention and control states are the reported results from the difference-in-differences models.

b *P* < .001.

c *P* < .05.

d Linear regression models included the following variables for controls: age, sex, Temporary Assistance for Needy Families recipient, months of private insurance coverage, State Children’s Health Insurance Program eligibility, and race/ethnicity.

## Discussion

We found that increasing provider reimbursements was an effective way to increase access to preventive care dental visits. Although tertiary services were not the intended target of our study, use of those services provides useful outcomes to assess long-term effects of increased preventive care visits. We hypothesized that increased preventive care dental visits would improve oral health to the degree that need for tertiary oral health services would be decreased. Our results partially supported this hypothesis by showing decreased dental emergency department visits. Increased reimbursements for preventive dental care were associated with increased sedation visits. Rather than an outcome for severe disease, sedation visits may indicate a population’s access to dental providers who diagnose and treat caries.

Although the overall frequency of emergency department visits increased over time in Texas, our model attributed a decline in these visits to a policy intervention to increase preventive care dental visits. Our findings support the idea that the use of emergency department visits is a sensitive indicator of a population’s lack of access to preventive dental care. Our findings provide a counterbalance to prior work on the association between declining reimbursements and increased ED visits for caries (25). Such visits for caries represent transient and ineffective care, because typical ED management is to address symptoms without addressing the disease (26). The relationship between timely access to preventive care and use of hospital services such as EDs has been established as a quality metric for medical conditions. The Agency for Healthcare Research and Quality created Prevention Quality Indicators (PQIs) (27), which measure quality of care for sensitive ambulatory care conditions. The rationale in developing PQIs was that certain conditions, when managed appropriately in the outpatient setting, can prevent severe exacerbations that warrant hospital services. PQIs provide a baseline for assessing the quality of health services at the population level and can be used to identify unmet needs (28). Although increased preventive care resulted in increased procedures to treat caries in our study, the decline in ED visits attributed to prevention quantifies the gap in a previously unmet need.

We were concerned about the potential for omitted variable bias, which would lead us to incorrectly attribute changes in use of dental service to a policy intervention that increased reimbursements for preventive dental care. To address this concern, we employed a difference-in-differences model with appendectomies as an outcome. We assumed that appendectomies were unlikely to be directly influenced by preventive dental care reimbursements. Although both dental and medical surgeries increased in association with increased dental reimbursements, only changes in appendectomies were found to be significant. We interpret the difference in significant change between medical versus dental surgical outcomes to further strengthen the validity of our findings that associate changes in use of dental care with increased reimbursements for preventive dental care. Had both dental and medical surgical outcomes changed in similar fashion, we would have concluded that our findings represented more general trends in health care use. Furthermore, a nonsignificant increase in dental surgeries of 0.01 percentage points with a baseline prevalence of 1.43% does not appear to be clinically meaningful at a population level. We interpret this to signify the limitations of an isolated policy intervention to increase access to preventive dental care on the oral health status of the dental surgery population.

Our study had limitations. First, budget constraints limited our study to only 1 year in our prepolicy period (2007) with a gap in data between the prepolicy and postpolicy period (2011–2012). Second, the ability to detect changes in disease burden was limited by use of the CDT coding system. The CDT coding system is an accurate system to track dental procedures, but it is an inadequate measure for the extent and severity of caries. Third, other possible sources of preventive dental care extend beyond dentists. State programs, such as North Carolina’s “Into the Mouths of Babes” (https://publichealth.nc.gov/oralhealth/partners/IMB.htm) have facilitated preventive dental care by nondental providers. However, because we defined preventive dental care as a claim for a service rather than specific to type of provider, our results reflect any preventive dental care, including that of primary care providers, reimbursed by Medicaid. Finally, we were unable to specify the mechanisms between increased provider reimbursements for prevention and use of tertiary oral health services. It has been demonstrated that reimbursement affects use of preventive services by expanding dental provider capacity, either by increasing the total number of participating providers or increasing the volume of patients seen by participating providers (12). We did not have access to data related to provider participation in state Medicaid programs, so we could not test for these relationships. Future work should address whether clinical management and treatment patterns change in response to an influx of Medicaid-enrolled children in a dental care delivery system.

Our findings suggest that a focus on other social determinants of oral health may be particularly influential in young children. The contribution of oral health behaviors, such as regular toothbrushing, restricted sugar intake, and exposure to fluoride may have greater impact than preventive care dental visits in families with young children who require dental surgery, particularly if these families do not seek care until after caries have developed. Future interventions may build on our findings by investigating the impact of multilevel interventions that address access to dental care as well as household oral health behaviors to change a population’s oral health status.
